# Paired Hierarchical Organization of 13-Lipoxygenases in *Arabidopsis*

**DOI:** 10.3390/plants5020016

**Published:** 2016-03-24

**Authors:** Adeline Chauvin, Aurore Lenglet, Jean-Luc Wolfender, Edward E. Farmer

**Affiliations:** 1 School of Pharmaceutical Sciences, University of Lausanne, University of Geneva, quai Ernest-Ansermet 30, CH-1211 Geneva 4, Switzerland; chauvinunige@gmail.com (A.C.); jean-luc.wolfender@unige.ch (J.-L.W.); 2 Department of Plant Molecular Biology, University of Lausanne, CH-1015 Lausanne, Switzerland; aurore.lenglet@unil.ch

**Keywords:** jasmonic acid, jasmonate, oxylipin, eicosanoid, wounding, defense, herbivore

## Abstract

Embryophyte genomes typically encode multiple 13-lipoxygenases (13-LOXs) that initiate the synthesis of wound-inducible mediators called jasmonates. Little is known about how the activities of these different LOX genes are coordinated. We found that the four *13-LOX* genes in *Arabidopsis thaliana* have different basal expression patterns. *LOX2* expression was strong in soft aerial tissues, but was excluded both within and proximal to maturing veins. *LOX3* was expressed most strongly in circumfasicular parenchyma. *LOX4* was expressed in phloem-associated cells, in contrast to *LOX6*, which is expressed in xylem contact cells. To investigate how the activities of these genes are coordinated after wounding, we carried out gene expression analyses in *13-lox* mutants. This revealed a two-tiered, paired hierarchy in which LOX6, and to a lesser extent LOX2, control most of the early-phase of jasmonate response gene expression. Jasmonates precursors produced by these two LOXs in wounded leaves are converted to active jasmonates that regulate *LOX3* and *LOX4* gene expression. Together with LOX2 and LOX6, and working downstream of them, LOX3 and LOX4 contribute to jasmonate synthesis that leads to the expression of the defense gene *VEGETATIVE STORAGE PROTEIN2* (*VSP2*). LOX3 and LOX4 were also found to contribute to defense against the generalist herbivore *Spodoptera littoralis*. Our results reveal that *13-LOX* genes are organised in a regulatory network, and the data herein raise the possibility that other genomes may encode LOXs that act as pairs.

## 1. Introduction

Lipoxygenases (LOXs) function to produce lipid mediators that operate in a broad range of processes, many of which are related to defense in animals [1] and in plants [2]. The *Arabidopsis thaliana* genome encodes six *LOX*s of which four are 13-LOXs, where “13” refers to the carbon atom in polyunsaturated 18-carbon fatty acids that are preferentially oxygenated by the LOX. 13-LOXs incorporate molecular oxygen into α-linolenic acid to produce its 13(*S*)-hydroperoxide [3], a molecule that is transformed into jasmonates which regulate wound-induced defense gene expression [4,5]. To complete jasmonate synthesis, fatty acid hydroperoxides formed through LOX action are dehydrated and cyclized to form the intermediate 12-oxo-phytodienoic acid (OPDA) in reactions catalysed by allene oxide synthase (AOS) and auxiliary proteins [6,7]. Further transformations of OPDA then result in the production of jasmonic acid (JA) and its biologically active derivatives, chief among which is jasmonyl-isoleucine (JA-Ile) [8].

Jasmonates (and/or their immediate precursors) produced in response to wounding do not stay where they are formed and can be transported efficiently within tissues. Following wounding, jasmonates/jasmonate precursors produced via LOX6 action in the vasculature of *Arabidopsis* leaves are highly mobile and move radially outwards from veins into the mesophyll [9]. In addition to jasmonate mobility, many 13-LOXs are themselves jasmonate-inducible. Therefore, in theory, jasmonates produced via the activity of any 13-LOX could be dispersed to different cell types capable of expressing other LOXs that also make jasmonate precursors. This raises an obvious question: how is the activity of the four *13-LOX* genes in *Arabidopsis* coordinated?

The roles of 13-LOXs in jasmonate-controlled defense responses have been studied in numerous plants, including, but not restricted to potato [10], wild tobacco [11], tomato [12], rice [13], and maize [14], as well as in *Arabidopsis*, a plant in which systematic *LOX* gene mutagenesis has been employed [15]. Intriguingly, while all four 13-LOXs encoded in the *A. thaliana* genome contribute to the synthesis of jasmonic acid [15], they each appear to have somewhat different functions in physically damaged leaves—the subject of the present work. For example, in addition to the initiation of JA synthesis in wounded leaves [16,17], LOX2 also plays a minor role in JA synthesis in undamaged leaves distal to wounds [18]. Furthermore, close to the site of damage, LOX2-derived hydroperoxides are also channelled into the synthesis of arabidopsides, galactolipids that carry one or more esterified OPDA or dinor-OPDA molecules [17,19,20,21]. Consistent with arabidopsides being defensive secondary metabolites, plants lacking LOX2 were more susceptible to the lepidopteran herbivore *Spodoptera littoralis* than is the wild type [17].

LOX6 also plays a role in leaf defense. The experiments that revealed this role involved the genetic removal of each of the three other 13-LOXs through producing a *lox2 lox3 lox4* triple mutant. In this plant, LOX6 functioning alone was capable of maintaining the defense of emerging leaves and shoot apical tissues in *Arabidopsis* rosettes [15]. Interestingly, the relative impact of LOX6 in early wound-stimulated jasmonate production in leaves increases with the distance from damage sites. That is, LOX6 was necessary for most of the rapid distal expression of the regulatory gene *JASMONATE ZIM-DOMAIN 10* (*JAZ10*) when the rosette was wounded [15], making this LOX of particular relevance in studies of long distance wound signalling.

Finally, the LOX3/LOX4 pair contributes approximately 20% of the total JA pool that accumulates in leaves in the first three minutes after wounding [15], however, no roles for these two LOXs in leaves are known. Here, we investigated the relative contributions of *LOX2, LOX3, LOX4*, and *LOX6* to each other’s expression, as well as to the expression of a defense gene. We then used herbivory assays to investigate LOX3 and LOX4 function in leaves. Our results revealed a lipoxygenase network that operates to coordinate jasmonate synthesis and defense responses in wounded leaves.

## 2. Results and Discussion

### 2.1. 13-LOX Expression Patterns in Unwounded Rosettes

**Figure 1:**
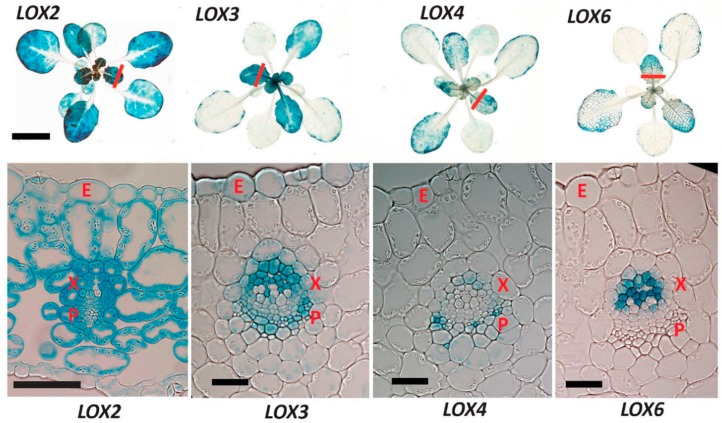
13-lipoxygenases (*13-LOX*) promoter-driven β-glucuronidase (GUS) activity in 3.5 week-old plants. Upper images: rosettes. Scale bar = 1 cm. Red bars indicate approximate section locations shown in lower images. E = epidermal cell; X = xylem region; P = phloem region. Scale bars for sections = 30 µm. Note that the *LOX2* section was cut nearer the leaf tip than the other *LOX* sections.

The expression patterns of *Arabidopsis* LOXs have been examined at the seedling stage [22], but equivalent data for leaves were lacking. Is each *13-LOX* expressed in a different leaf cell type? To characterize basal *13-LOX* gene activity in unwounded leaves, each promoter was fused to a secretable β-glucuronidase (*GUS*) reporter gene. LOX6, principally expressed in xylem contact cells [9,15], served as a comparison with other *13-LOX*s, as shown in Figure 1. GUS staining in younger leaves was stronger than in older leaves for all *13*-*LOX* promoters, and sections of younger leaves were compared. *LOX2* had the only promoter among the four that was widely active in most tissues except in and near maturing veins. Because of this, transversal sections for visualizing *LOX2* reporter expression were cut nearer the leaf tip than for the other reporters (red bars in Figure 1). LOX2 protein is readily detectable in leaves [23] and *LOX2* expression was strong in mesophyll cells, bundle sheaths, and leaf-tip vasculature—but only at a distance from maturing veins. *LOX3* activity was perivascular and strongest in the xylem region, with weaker expression in the mesophyll. *LOX4* promoter activity was strongest in small cells in the phloem region. These might be companion cells, although their identity was not verified. Phloem is a known region of JA synthesis enzyme localisation [24]. We noted that *LOX2* expression was almost a “mirror image” of the *LOX3*, *LOX4*, and *LOX6.* The expression of these three *LOX*s (unlike for *LOX2*) was strong in the maturing vasculature of expanding leaves.

**Figure A1:**
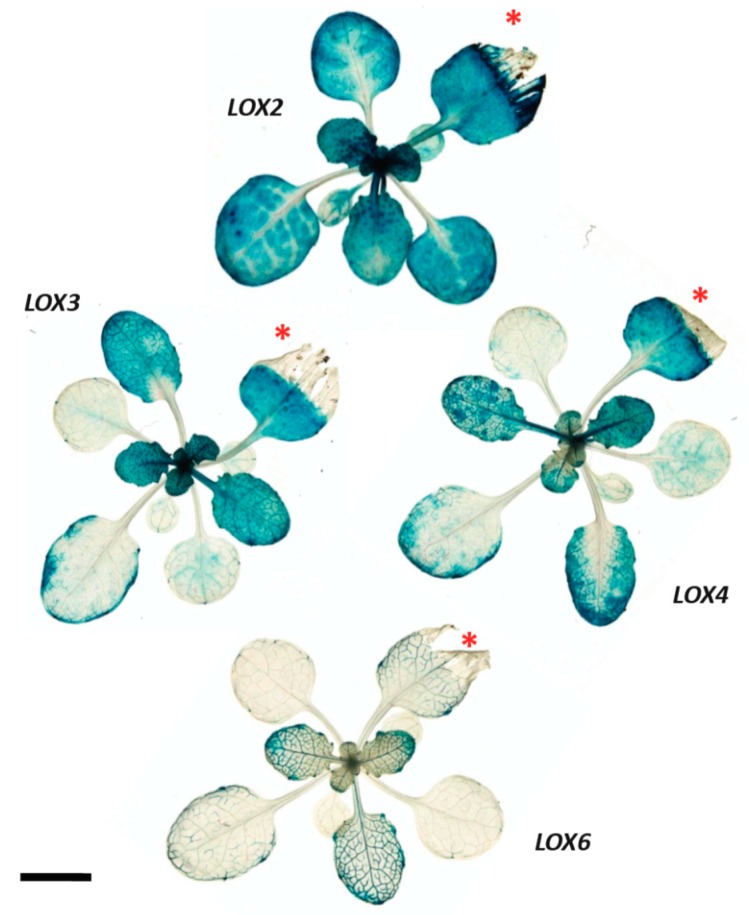
Expression of *LOX* promoter::GUS reporters in wounded rosettes. Plants were wounded on a single leaf (orange asterisk) and harvested 6 h later prior to staining for GUS activity. Scale bar = 1 cm.

Notably, all 13-LOXs are expressed in vascular tissues with only basal *LOX2* expression excluded from maturing veins. *LOX3* and *LOX6* expression is strongest near the xylem. *LOX4* is the only *13-LOX* to show basal expression almost exclusively in the phloem region. In terms of cellular space covered by promoter activities, *LOX4* and *LOX6* in unwounded leaves display a relatively small basal promoter activity domain, whereas the other two *13-LOXs* (*LOX2* and *LOX3*) have more extensive basal activity domains (Figure 1). GUS staining in the rosettes of wounded plants is shown in the Appendix (Figure A1).

### 2.2. LOX2 and LOX6 Regulate LOX3 and LOX4 Expression

**Figure 2:**
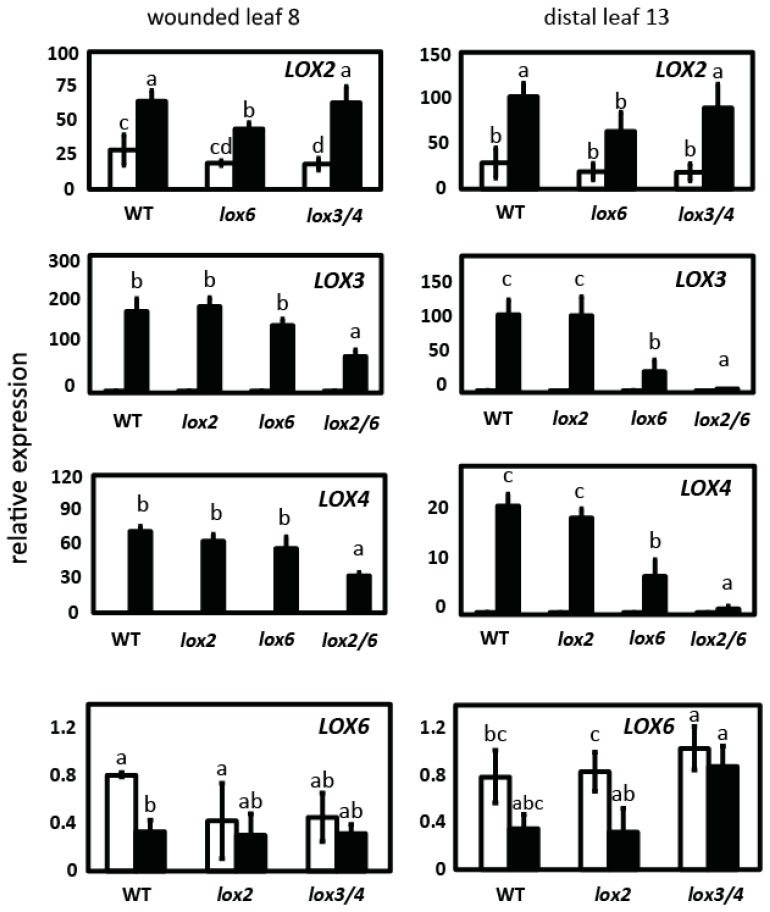
*13-LOX* gene expression in different *13-lox* mutant backgrounds. *LOX2* expression analysed in WT, in *lox6A* and in *lox3B lox4A* double mutants. *LOX3* and *LOX4* expression was analyzed in WT, *lox2-1*, and *lox6A* single mutants, and the *lox2-1 lox6A* double mutant. *LOX6* expression was analyzed in *lox2-1* and the *lox3B lox4A* double mutant. Leaves 8 (wounded) and 13 (distal) were snap-frozen before (unfilled bars) and 1 h after the wounding (filled bars). Data are from three (controls) and three to four (wounded) biological replicates (±SD). Letters (a, b, and c) refer to significant differences (ANOVA and *t*-test; *p* < 0.05).

**Figure A2:**
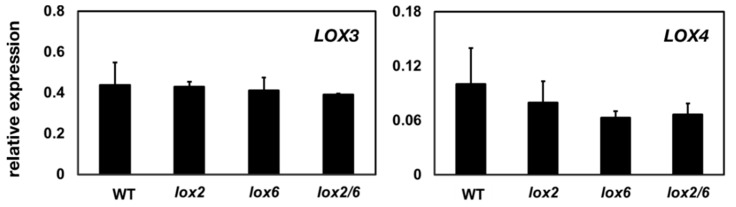
Basal *LOX3* and *LOX4* expression is not affected in the *lox2* and *lox6* backgrounds. Samples were from leaf 8 of five-week-old plants. *LOX3* and *LOX4* transcript levels are relative to those of *UBC21*.

*LOX6* transcripts are not wound-inducible in roots [25] or leaves [9,15]. *LOX2* expression was investigated in the *lox6* single mutant and the *lox3 lox4* double mutant. *LOX2* remained wound-inducible in each of the genetic backgrounds, however, in the absence of the functional *LOX6* gene, there was weakly reduced *LOX2* expression following wounding (Figure 2). LOX6 activity therefore contributes to *LOX2* gene expression in wounded leaves. The possibility that there are compensatory effects whereby above-WT activity of a particular *13-LOX* gene is stimulated by mutation of one or more of its homologues was tested. Figure A2 shows that basal *LOX3* or *LOX4* expression was not affected in unwounded leaves in the *lox2*- and *lox6*-containing backgrounds.

*LOX3* and *LOX4* transcript levels in the WT and both *lox2* and *lox6* single mutants were similar in the wounded leaf, while *LOX6* was found to be required for full, wound-induced *LOX3* and *LOX4* transcript levels in the distal leaf (Figure 2). In the wounded leaf, the double mutant *lox2 lox6* displayed 2-fold lower inductions of *LOX3* and *LOX4* transcripts compared to the WT. In the distal leaf, *LOX3* and *LOX4* gene expression was reduced by 97.2% and 96.3%, respectively, in the *lox2 lox6* double mutant relative to the WT. In the distal leaf, the *lox6* single mutant displayed an approximately 20-fold induction of *LOX3* transcripts, but in *lox2* these transcripts were induced to a far higher level (approximately 100-fold)—that is to a level similar to that in the WT. Similarly, a strong effect on distal *LOX4* transcript accumulation was observed in *lox6* compared to *lox2*. *LOX6* was not wound-inducible in the WT or in *lox* mutant backgrounds (Figure 2). The low transcript levels and associated large error bars for this *LOX* limit the interpretation of this result.

### 2.3. 13-LOXs Contribute Differentially to Inducible VSP2 Defense Gene Expression

**Figure 3:**
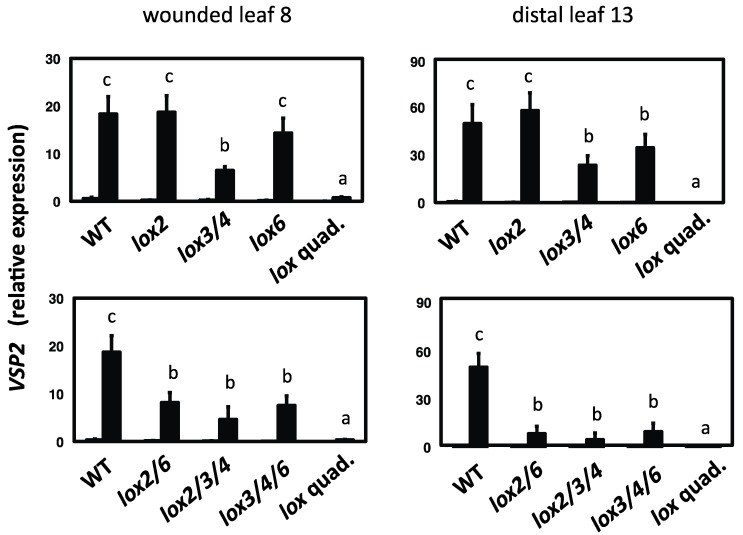
Wound-induced *VSP2* expression in *13-lox* mutants. Leaves 8 (wounded) and 13 (distal) were snap-frozen at 4 h after wounding (filled bars) or harvested at the same timepoint from unwounded plants (unfilled bars). Data from three (unwounded controls) to four (wounded samples) biological replicates (±SD). Letters (a, b, and c) refer to significant differences (ANOVA and t-test; *p* < 0.05). *VSP2* transcript levels in unwounded plants is low and statistics are shown for wounded treatments only. WT = wild type; *lox2* = *lox2-1*; *lox3/4* = the *lox3B lox4A* double mutant; *lox6* = *lox6A*; *lox2/6* = the *lox2-1 lox6A* double mutant; *lox2/3/4* = *lox2-1 lox3B lox4A* triple mutant; *lox3/4/6* = *lox3B lox4A lox6A* triple mutant; *lox* quad. = *lox2-1 lox3B lox4A lox6A* quadruple mutant.

The expression of the jasmonate-regulated defense gene (*VEGETATIVE STORAGE PROTEIN2; VSP2*) was then investigated. *VSP2* is expressed at maximal levels several hours after wounding leaves [26] so a 4 h timepoint after wounding was chosen for initial experiments. *VSP2* transcript levels after wounding were reduced relative to the WT in the wounded leaf of the double mutant *lox3 lox4*, while in the distal leaf their levels were reduced in both the *lox3 lox4* double mutants and the *lox6* single mutant (Figure 3). Consistent with a role of all 13-LOXs in controlling *VSP2* expression through jasmonate production, the *lox2 lox6* double mutant, and the *lox2 lox3 lox4* and *lox3 lox4 lox6* triple mutants were unable to reach WT *VSP2* levels in either the wounded leaf or the distal undamaged leaf. Figure 3 shows that *VSP2* expression was significantly reduced in the *lox2 lox6* double mutant, whereas the *lox6* mutation alone had little effect on *VSP2* transcript levels.

### 2.4. Comparison of LOX3, LOX4 and VSP2 Expression in the WT and the lox2 lox6 Double Mutant

**Figure 4:**
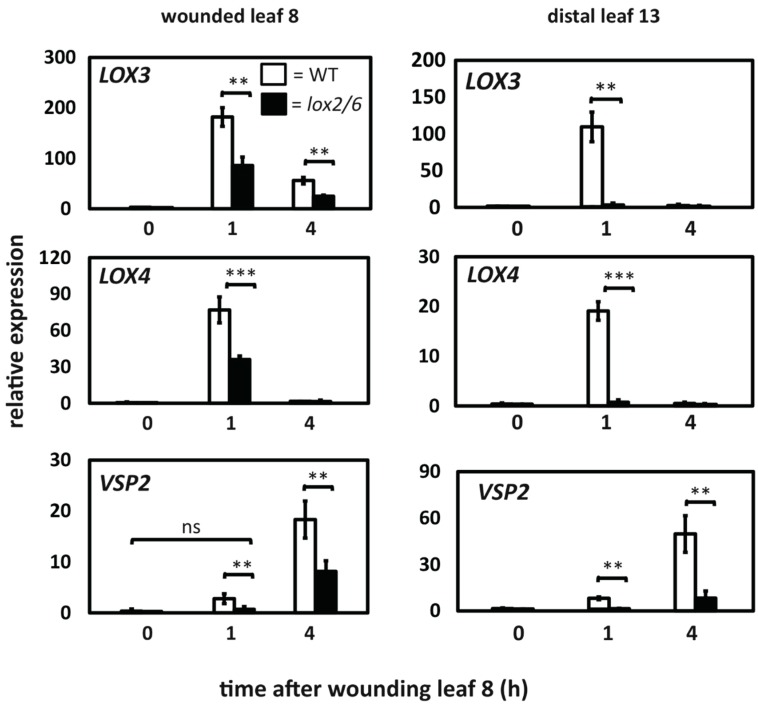
Wound-inducible *LOX3*, *LOX4*, and *VSP2* expression in WT (open bars) and the *lox2-1 lox6A* double mutant (black bars). Leaves 8 (wounded) and 13 (distal) were snap-frozen before and after wounding. Data are from three to four biological replicates (±SD). Data from a 2h timepoint also included in the original experiment are not shown. Asterisks refer to data significantly different from WT (ns: not significant; *, *p* < 0.05; **, *p* < 0.01, and ***, *p* < 0.001; *t*-test).

To gain insights into the temporal control of gene expression we compared the wound induction of *LOX3*, *LOX4*, and *VSP2* transcripts in the WT and the *lox2 lox6* double mutant. As shown in Figure 4, *LOX3* and *LOX4* transcript levels were upregulated rapidly (within 1 h) in the wounded leaf and in the distal leaf of the WT. However, *LOX3* and *LOX4* transcript accumulation in response to wounding was reduced in the *lox2 lox6* double mutant. The possibility that the *lox2 lox6* mutations might cause increased expression of *LOX3* and *LOX4* is, given the results in Figure 2, considered unlikely. Over the experimental period, *LOX3* and *LOX4* transcript levels in the distal leaf of *lox2 lox6* never reached WT levels. This is consistent with the LOX2/LOX6 pair acting upstream of the LOX3/LOX4 pair and contributing to their expression. Using an identical timeframe we observed that *VSP2* transcripts accumulated with different kinetics in the WT and the *lox2 lox6* double mutant. The wound-induced expression of *VSP2* was reduced in the *lox2 lox6* double mutant (*i.e.*, LOX2 and LOX6 are essential for full *VSP2* gene expression). This led us to test the roles of the *LOX3/LOX4* gene pair in herbivory assays.

### 2.5. All 13-LOXs Act in Defense against a Lepidopteran Herbivore

**Figure 5:**
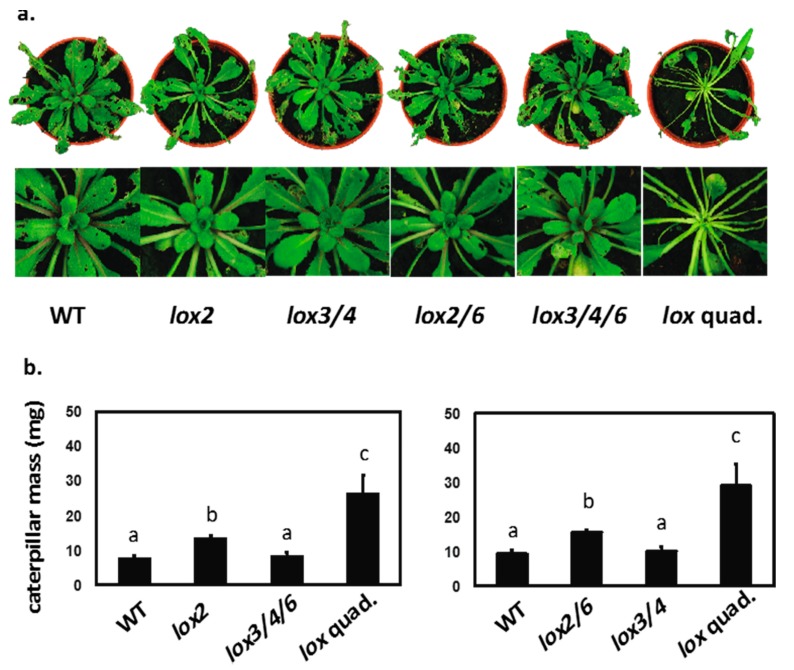
Contributions of 13-LOXs to defense against a chewing herbivore. (**a**) Damage to rosettes inflicted by *Spodoptera littoralis* larvae. WT = wild type; *lox3/4* = the *lox3B lox4A* double mutant; *lox2/6* = the *lox2-1 lox6A* double mutant; lox3/4/6 = *lox3B lox4A lox6A* triple mutant; lox quad. = *lox2-1 lox3B lox4A lox6A* quadruple mutant; (**b**) Larval mass after feeding. Insects were harvested at 11d. Letters (a, b, and c) refer to significant differences (ANOVA and t-test; *p* < 0.05).

The *lox2 lox3 lox4 lox6* quadruple mutant is known to display greatly reduced resistance to larvae of *Spodoptera littoralis* relative to the WT [15]. This genotype was used as a control to investigate the roles of *LOX2*, *LOX3*, and *LOX4* in defense. In all plants—except the quadruple mutant, where the center of the rosette was attacked—damage inflicted by *S. littoralis* was restricted to expanded leaves (Figure 5a). Larvae also grew fast on the *lox2-1* single mutant (Figure 5b) as described in Glauser *et al.* [17]. To further investigate LOX2 function we used the LOX2 “mirror” mutant (*i.e.*, the triple mutant *lox3 lox4 lox6* that retains functional LOX2 as its only 13-LOX). As judged by measuring insect weight gain, *LOX2* on its own in the triple mutant mediated near-WT-levels of defense (Figure 5a,b). Since redundancy between *LOX3* and *LOX4* has been observed in early responses to leaf wounding [15], larval growth was examined on a *lox3 lox4* double mutant and on the “mirror” mutant *lox2 lox6* that retains functional LOX3 and LOX4 as its only 13-LOXs. Caterpillar growth was found to be similar to that on the WT for both the *lox3 lox4* double and *lox3 lox4 lox6* triple mutants. However, the insects gained 1.7 times more weight than they did on the WT when feeding on the *lox2 lox6* double mutant, but this weight gain was less than that seen on the quadruple mutant (3.1 times more weight gain than on the WT). That is, the presence of functional *LOX3* and *LOX4* in the *lox2 lox6* double mutant actively reduced insect weight gain.

In summary, while *LOX2* and *LOX6* are known to make different contributions to leaf defense [9,15], the new results show that *LOX3* and *LOX4* also help protect the rosette.

### 2.6. Functional Pairs of 13-LOXs in Leaf Defense

**Figure 6:**
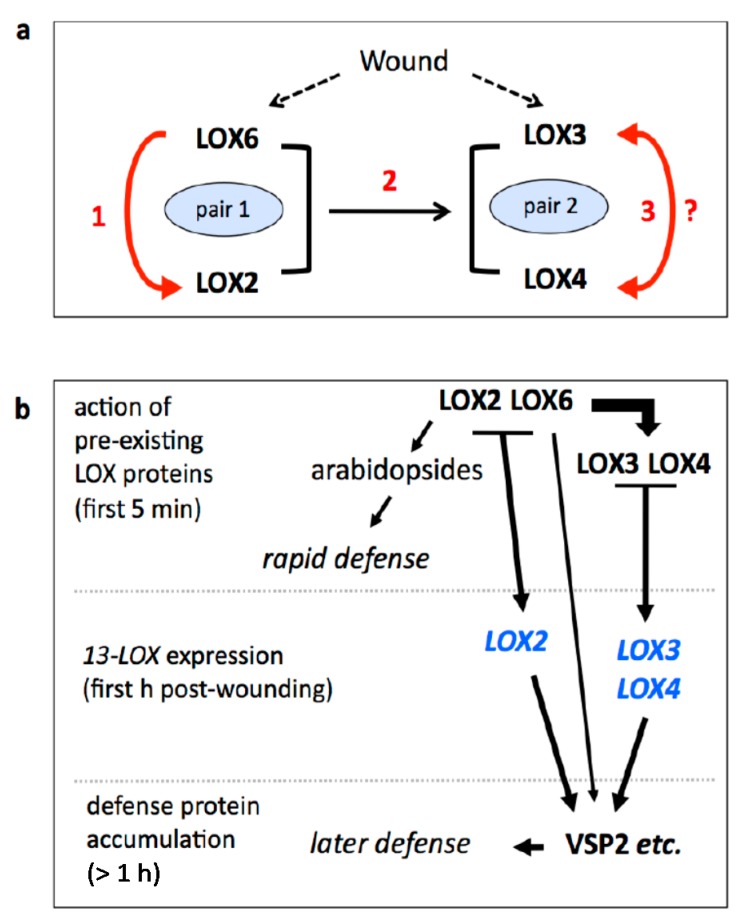
Figure 6. 13-LOX activities in wounded *Arabidopsis* leaves. (a) 13-LOXs interact through jasmonate production. 1. LOX6 participates in the production of jasmonate that upregulates *LOX2* expression (from Figure 2). 2. LOX6 and LOX2 together produce jasmonates that enhance *LOX3* and *LOX4* expression (from Figure 2). 3. Still hypothetical, LOX3 and LOX4 may enhance each other’s expression through jasmonate production; (b) 13-LOX roles in the wound response. Preformed LOX2 rapidly produces arabidopsides, secondary compounds implicated in direct defense [17]. LOX6 and LOX2 produce jasmonates necessary for early gene expression (e.g., *JAZ10*; 9,15 and *LOX3/4* expression; Figure 2 and Figure 4). LOX3 and LOX4 produce jasmonates that ensure correct late-phase *VSP2* gene expression (from Figure 3 and Figure 4). Proteins, black; transcripts, blue.

An outcome of the present work was the finding that 13-LOX activities appear to function in an organised regulatory network. Specifically, we provide evidence consistent with LOX2 and LOX6 functioning upstream of LOX3 and LOX4. This is shown in Figure 6.

While we suggest that LOX2/LOX6 and LOX3/LOX4 act in pairs in *Arabidopsis*, it is important to note that each enzyme has some distinct roles. LOX2 activity in the Columbia (Col) accession is dedicated in large part to the rapid production of arabidopsides in and near wounds [17]. LOX2 is closely related to a *Nicotiana attenuata* LOX that produces substrates for 2(*E*)-hexenal synthesis via HPL activity [27]. However, the *HPL* (*HYDROPEROXIDE LYASE*) gene, which uses 13-LOX products for the synthesis of volatile aldehydes, is mutated in Col accessions—and Col plants produce little or no 2(*E*)-hexenal upon wounding [28]—so it is possible that LOX2 would also produce this volatile in accessions with a functional *HPL* gene. Moreover, *LOX2* expression seems to be optimally placed for the defense of soft tissues: it would be difficult for a chewing herbivore to avoid damaging cells in its broad basal expression domain. As shown both previously [9] and herein, LOX2 also contributes to the production of biologically active jasmonates in leaves distal to wounds, and these jasmonates stimulate *LOX3*, *LOX4*, and *VSP2* expression.

LOX6 produces the precursors of biologically active jasmonates, and it is possible that this enzyme builds pre-formed OPDA pools that are mobilized on wounding [25]. The relative level of jasmonate originating from LOX6 activity increases at a distance from a wound [15], and LOX6 plays a more powerful role than LOX2 in this respect. LOX6, like LOX2, contributes to *VSP2* gene expression. Finally, *lox6* mutants are more drought-sensitive than the WT, hinting at other roles for this LOX [25]. LOX3 and LOX4 proteins also contribute to jasmonate synthesis that then leads to increased *LOX3*, *LOX4*, and *VSP2* expression. Alone (*i.e.*, in the absence of LOX6 and LOX2) the expression of the *LOX3/LOX4* pair is barely activated in leaves distal to wounds (note that it is possible that long-distance wound signals activate pre-formed LOX3 and LOX4 enzymes). However, *LOX3* transcript levels increase after mechano-stimulation [29], and LOX3 protein is jasmonate-inducible in the vicinity of wounds [30]. Under the conditions used herein, LOX3 and LOX4 do not strongly activate *LOX2* and *LOX6* gene expression (Figure 2), therefore arrow 2 in Figure 6**a** is unidirectional. Our studies have not determined the relative contributions of LOX3 and LOX4 to leaf defense, however, differential activities for these LOXs in leaves are possible [31]. Work remains to further refine the models shown in Figure 6. Additionally, the LOX network we propose is likely to extend beyond 13-LOXs and there is already evidence for interaction between 13- and 9-LOX pathways in rice (e.g., [32]). In *Arabidopsis*, transcripts of *LOX1*, a 9-lipoxygenase, are strongly jasmonate-inducible [33], raising the possibility that the 13-LOX network encompasses 9-LOXs.

Lastly, each of the four *Arabidopsis* 13-LOXs should provide unique insights into signalling mechanisms that operate to initiate jasmonate synthesis. Recently, it was proposed that stress-responsive signal pathways involving coupled glutamate receptor-like/lipoxygenase (GLR/LOX) modules may exist [34]. If so, each GLR-LOX pathway might have unique characteristics.

## 3. Experimental Section

### 3.1. Plant Material and Growth Conditions

*A. thaliana* (L.) Heynh. T-DNA insertion mutants in the Columbia background were obtained from the European Arabidopsis Stock Center (NASC): *LOX3* (At1g17420; *lox3B* = SALK_147830), *LOX4* (At1g72520; *lox4A* = SALK_071732), and *LOX6* (At1g67560; *lox6A* = SALK_138907). The *lox2-1* (At3g45140) mutant, double, triple, and quadruple mutants all based on the *lox2-1, lox3B, lox4A*, and *lox6A* alleles have been described previously [15,17,35]. The *aos* mutant was from [36]. Plants were grown individually in 7 cm diameter pots at 21 °C, 10 h light d^−1^ (100 μmol·s^−1^·m^−2^) photoperiod, and 70% humidity. Leaves of five-week-old plants were numbered from oldest to youngest. All wounding experiments (crushing 50% of leaf 8 with metal forceps) were performed between 12 a.m. and 16.30 p.m. Leaves were snap-frozen in liquid N_2_ and stored at −80 °C before extractions for RT-qPCR.

### 3.2. Insect Feeding Assays

Eleven pots, each with a 4.5 week-old plant, were isolated in eleven plexiglass boxes. Newly hatched *Spodoptera littoralis* (Boisduval; Noctuidae; Lepidoptera) caterpillars were placed on each plant (four larvae per pot). Larvae were harvested after 11 days. The weight of larvae from one box per number of recovered larvae was considered as one biological replicate [15].

### 3.3. Gene Expression

RT-qPCR was performed as described in [15] with a PCR program detailed in [30]. Data were standardized to the *UBC21* ubiquitin conjugase reference gene. The following primers were used: *UBC21* (At5g25760): 5′-CAGTCTGTGTGTAGAGCTATCATAGCAT-3’, 5′-AGAAGATTCCCTGAGTCGCAGTT-3’. *VSP2* (At5g24770): 5’-CATCATAGAGCTCGGGATTGAACCC-3’, 5’-AGATGCTTCCAGTAGGTCACGC-3’. *LOX2*: 5’-ATTACGGTAGAAGACTACGCACAAC-3’, 5’-GTAATTTAAGCTCTACCCCCTTGAG-3’. *LOX3*: 5’-AACACAACCACATGGTCTTAAACTC-3’, 5’-GGAGCTCAGAGTCTGTTTTGATAAG-3’. *LOX4*: 5’-ATAGAACGAGTCACGTCTTATCCAC-3’, 5’-CATAAACAAACGGTTCGTCTCTAAC-3’.

### 3.4. GUS Staining and Light Microscopy

Promoter/GUS fusion plants for *LOX2* (At3g45140), *LOX3* (At1g17420), and *LOX4* (At1g72520) were from ([9]; see supplemental material in that paper for LOX3_pro_:GUS and LOX4_pro_:GUS). The *LOX6*_pro_:GUS fusion plants were from [15]. 26 individual T_1_ plants from each construct were stained and at least six selected to produce T_2_ independent lines from which at least six for each independent line were stained for in-depth analysis. For staining and embedding [37], 3.5 week-old rosettes were prefixed in acetone:water (90:10, v:v) for 45 min on ice, washed twice in 50 mM sodium phosphate buffer (pH 7.2), and vacuum-infiltrated for 10 min (for rosette staining) or 2 h (for leaf sectioning), then left 16 h at 37 °C in the dark in 10 mM Na_2_EDTA; 50 mM sodium phosphate buffer, pH 7.2; 2 mM (for rosettes) or 3 mM (for thin sections) K_4_Fe(CN)_6_; 2 mM (for rosettes) or 3 mM (for thin sections) K_3_Fe(CN)_6_; 0.1% (v/v) Triton X-100; 0.6 mg·mL^−1^ 5-bromo-4-chloro-3-indolyl-β-d-glucuronide (X-Gluc). Rosettes were then either transferred to ethanol: water (70:30, v/v) for photography or fixed (glutaraldehyde:formaldehyde:50 mM sodium phosphate (pH 7.2) buffer = 2:5:43, v:v:v). For embedding, stained leaves were dehydrated in an ethanol gradient (10%, 30%, 50%, 70%, 90%, and twice absolute) for 30 min. Embedding in Technovit 7100 resin (Haslab GmbH, Ostermundigen, Switzerland) was carried out according to the manufacturer’s instructions. Sections (5 μm) were made with a RM2255 microtome (Leica, Muttenz, Switzerland) using disposable Leica TC-65 blades.

## 4. Conclusions

Our results highlight the fact that three of the four *13-LOX*s in *Arabidopsis* are expressed primarily in the vasculature. The exception, *LOX2*, is expressed in soft tissues; this *LOX* has only low basal expression in mature veins. Although they have different roles in the wound response, all four *13-LOX*s operate together in a regulatory network. This network can be seen as being comprised of two pairs of *LOX*s; the first pair (LOX6 and LOX2) produces precursors of jasmonates in response to wounding. This helps to control the expression of the second *LOX* pair: *LOX3* and *LOX4*, and the enzymes encoded by these genes also contribute to jasmonate synthesis. We raise the possibility that other plant genomes may encode pairs of *LOX*s, and that these may also act hierarchically to control each other’s expression.
